# IL-10 and IL-12 (P70) Levels Predict the Risk of Covid-19 Progression in Hypertensive Patients: Insights From the BRACE-CORONA Trial

**DOI:** 10.3389/fcvm.2021.702507

**Published:** 2021-07-27

**Authors:** Renata Moll-Bernardes, Andrea Silvestre de Sousa, Ariane V. S. Macedo, Renato D. Lopes, Narendra Vera, Luciana C. R. Maia, André Feldman, Guilherme D. A. S. Arruda, Mauro J. C. Castro, Pedro M. Pimentel-Coelho, Denílson C. de Albuquerque, Thiago Ceccatto de Paula, Thyago A. B. Furquim, Vitor A. Loures, Karla G. D. Giusti, Nathália M. de Oliveira, Fábio A. De Luca, Marisol D. M. Kotsugai, Rafael A. M. Domiciano, Mayara Fraga Santos, Olga Ferreira de Souza, Fernando A. Bozza, Ronir Raggio Luiz, Emiliano Medei

**Affiliations:** ^1^D'Or Institute for Research and Education, Rio de Janeiro, Brazil; ^2^Evandro Chagas National Institute of Infectious Disease, Oswaldo Cruz Foundation, Rio de Janeiro, Brazil; ^3^Hospital São Luiz Jabaquara, São Paulo, Brazil; ^4^Santa Casa de São Paulo, São Paulo, Brazil; ^5^Duke Clinical Research Institute, Duke University Medical Center, Durham, NC, United States; ^6^Brazilian Clinical Research Institute, São Paulo, Brazil; ^7^Institute of Biophysics Carlos Chagas Filho, Federal University of Rio de Janeiro, Rio de Janeiro, Brazil; ^8^Hospital São Luiz Anália Franco, São Paulo, Brazil; ^9^Hospital São Luiz São Caetano, São Caetano do Sul, Brazil; ^10^Instituto de Microbiologia Paulo de Góes, Federal University of Rio de Janeiro, Rio de Janeiro, Brazil; ^11^Cardiology Department, Rio de Janeiro State University, Rio de Janeiro, Brazil; ^12^Hospital Sino Brasileiro, Osasco, Brazil; ^13^Hospital Villa Lobos, São Paulo, Brazil; ^14^Hospital São Luiz Morumbi, São Paulo, Brazil; ^15^Hospital Copa Star, Rio de Janeiro, Brazil; ^16^Institute for Studies in Public Health—IESC, Federal University of Rio de Janeiro, Rio de Janeiro, Brazil; ^17^National Center for Structural Biology and Bioimaging, Federal University of Rio de Janeiro, Rio de Janeiro, Brazil

**Keywords:** hypertension, cytokine, COVID-19, biomarker, inflammation, prognosis

## Abstract

**Background:** Cardiovascular comorbidities such as hypertension and inflammatory response dysregulation are associated with worse COVID-19 prognoses. Different cytokines have been proposed to play vital pathophysiological roles in COVID-19 progression, but appropriate prognostic biomarkers remain lacking. We hypothesized that the combination of immunological and clinical variables at admission could predict the clinical progression of COVID-19 in hypertensive patients.

**Methods:** The levels of biomarkers, including C-reactive protein, lymphocytes, monocytes, and a panel of 29 cytokines, were measured in blood samples from 167 hypertensive patients included in the BRACE-CORONA trial. The primary outcome was the highest score during hospitalization on the modified WHO Ordinal Scale for Clinical Improvement. The probability of progression to severe disease was estimated using a logistic regression model that included clinical variables and biomarkers associated significantly with the primary outcome.

**Results:** During hospitalization, 13 (7.8%) patients showed progression to more severe forms of COVID-19, including three deaths. Obesity, diabetes, oxygen saturation, lung involvement on computed tomography examination, the C-reactive protein level, levels of 15 cytokines, and lymphopenia on admission were associated with progression to severe COVID-19. Elevated levels of interleukin-10 and interleukin-12 (p70) combined with two or three of the abovementioned clinical comorbidities were associated strongly with progression to severe COVID-19. The risk of progression to severe disease reached 97.5% in the presence of the five variables included in our model.

**Conclusions:** This study demonstrated that interleukin-10 and interleukin-12 (p70) levels, in combination with clinical variables, at hospital admission are key biomarkers associated with an increased risk of disease progression in hypertensive patients with COVID-19.

## Introduction

COVID-19 may evolve to severe viral pneumonia and acute respiratory distress syndrome with a high mortality rate. Importantly, patients with cardiac comorbidities have been found in various studies to be at greater risk of severe disease (1–6). In addition, patients with cardiovascular disease are more prone to myocardial injury development after SARS-CoV-2 infection (7–9).

The pathophysiological mechanisms related to these increased risks in patients with cardiac comorbidities are not completely understood. Concern has been raised about the use of angiotensin-converting enzyme inhibitors (ACEIs) and angiotensin receptor blockers (ARBs) in hypertensive patients, as preclinical studies have suggested that renin-angiotensin-aldosterone system inhibitors increase the expression of angiotensin-converting enzyme 2, the functional SARS-CoV-2 receptor (10–12). A recent randomized trial from our group (the BRACE-CORONA trial), in which 659 hypertensive patients were included, demonstrated that the discontinuation of ACEIs and ARBs for 30 days does not impact the number of days over a 30-day follow-up period that patients hospitalized with mild to moderate COVID-19 remain alive and out of the hospital (13, 14).

In addition to cardiac risk factors, several studies have suggested the occurrence of a dysregulated inflammatory response, characterized by the simultaneous release of pro- and anti-inflammatory mediators, known as a cytokine storm and established as a key factor in the physiopathology and clinical progression of COVID-19 in a subset of patients (15, 16). An exacerbated immune response is well-accepted to potentially strongly impair cardiac function (17–20). Several cytokines have been proposed to be potential biomarkers of COVID-19 severity (21–24); interferon gamma–induced protein 10 (IP-10), interleukin (IL)-6, and IL-10 have been associated consistently with greater severity of this disease (25–28).

The uncertainty and variability of the innate immune response, associated with an unpredictable disease course ranging from mild to fatal, highlights the need to identify prognostic factors related to a greater risk of progression to severe disease, particularly in more susceptible patients with comorbidities such as hypertension. To our knowledge, however, no data have been provided about biomarkers that could allow clinicians to identify, in the first 48 h after hospital admission, hypertensive patients at increased risk of disease progression, thereby helping them to choose the best therapeutic option.

This study was conducted to test the hypothesis that the immunological profiles of hypertensive patients upon admission to hospital with COVID-19 provide additional information about disease severity and progression. The analysis of cellular components, such as lymphocytes and monocytes, and the quantification of cytokine concentrations were performed to identify potential biomarkers.

## Materials and Methods

### Population and Design

Patients included in this study were from the BRACE-CORONA trial (14), an academically led, investigator-initiated phase IV multicenter open-label registry-based randomized trial involving 659 patients on ACEIs/ARBs with confirmed COVID-19 diagnoses at 29 centers in Brazil. The present study was conducted with blood samples from 167 hospitalized hypertensive patients enrolled consecutively in the trial at six centers in the state of São Paulo, Brazil. The samples were collected within 24 h of COVID-19 diagnosis confirmation between 21 May and 27 June 2020. The trial protocol (13) was approved by the Brazilian Ministry of Health National Commission for Research Ethics and by institutional review boards or ethics committees at participating sites. All patients provided informed consent before enrollment.

Patients eligible for the BRACE-CORONA trial were aged ≥18 years and chronic ACEI/ARB users. Patients with clinical indications for ACEI/ARB treatment termination, such as hypotension, acute kidney injury, and/or shock, were excluded. Patients on mechanical ventilation and those with hemodynamic instability, acute renal failure, or shock also were excluded (14). The inclusion and exclusion criteria are provided in full in the Supplementary Data.

### Outcomes

The primary outcome was defined as the highest score during hospitalization on the modified WHO Ordinal Scale for Clinical Improvement [range, 0 (no evidence of infection) to 8 (death)]. COVID-19 was classified as non-severe (mild to moderate, scores of 3–5), ranging from the lack of need for oxygen therapy to conditions requiring noninvasive ventilation, and severe (scores of 6–8), including disease necessitating the use of mechanical ventilation, inotropic support, and/or renal replacement therapy, and that causing death (Supplementary Table 1) (29). Secondary outcomes were the lengths of stay (LOSs) in the hospital and intensive care unit (ICU), acute myocardial infarction, new or worsening heart failure, hypertensive crisis, transient ischemic attack, stroke, myocarditis, pericarditis, arrhythmias requiring treatment, and thromboembolic events.

### Biomarker Quantification

Blood samples were collected in tubes containing ethylenediaminetetraacetic acid as an anticoagulant and centrifuged immediately for plasma separation. Plasma samples were then frozen and stored at −20°C until analysis. Levels of epidermal growth factor, eotaxin, granulocyte colony-stimulating factor (G-CSF), granulocyte-macrophage colony-stimulating factor (GM-CSF), interferon (IFN)-α2, IFN-γ, IL-1α, IL-1β, IL-1ra, IL-2–8, IL-10, IL-12 (p40), IL-12 (p70), IL-13, IL-15, IL-17A, IP-10, monocyte chemoattractant protein-1 (MCP-1), macrophage inflammatory protein (MIP)-1α, MIP-1β, tumor necrosis factor (TNF)-α, TNF-β, and vascular endothelial growth factor in undiluted samples were measured using the MILLIPLEX MAP human cytokine/chemokine magnetic bead panel (HCYTMAG-60K-PX29; Merck Millipore, Billerica, MA, USA) according to the manufacturer's instructions. The assay plates were read immediately and analyzed in a MAGPIX® system (Merck Millipore). All samples and standards were measured in duplicate. Lymphocyte and monocyte quantification was performed in an automized Horiba ABX Micros 60 system (Horiba Medical, Montpellier, France) using photometry. C-reactive protein (CRP) was measured by latex-enhanced immunoturbidimetric assay. Cytokines not detected in >50% of the patient samples were excluded from further analyses.

### Statistical Analysis

Continuous variables were described as medians, means, and standard deviations; categorical variables were characterized by proportions. For the primary outcome, 95% confidence intervals (CIs) were calculated. Fisher's exact test was used to detect statistical associations between the outcome and categorical clinical variables. For continuous variables, receiver operating characteristic (ROC) curves were used to discriminate between severe and non-severe cases, and those associated statistically with the primary outcome were dichotomized using cutoff points of 90% sensitivity. *P* ≤ 0.05 was used to define significance and for automatic forward stepwise selection of clinical variables for inclusion in a binary logistic regression model. The significance levels for entry and removal of variables selected by the automatic regression model were defined at 5 and 10%, respectively. The beta coefficients and odd ratios were calculated for all variables in each step of the model to quantify the association with the outcome. The goodness of fit for the final model was evaluated by the Hosmer–Lemeshow test and by ROC curve. Predicted probabilities for the primary outcome were estimated using variables showing significant associations in the final model. All analysis were performed using SPSS software (version 24.0; IBM Corporation, Armonk, NY, USA).

## Results

Of the 167 hypertensive patients, 13.8% were using ACEIs and 86.2% were using ARBs. The mean patient age was 54.1 ± 12.3 years; 57 (34.1%) patients were female, 88 (52.7%) were obese, 41 (24.6%) had diabetes, and 29 (17.4%) had dyslipidemia. Coronary artery disease and chronic pulmonary disease were present in 2.4% of the cases each, and 2.4% of the patients were smokers. Data on all comorbidities are provided in Supplementary Figure 1.

Cough (62.3%), fever (57.5%), myalgia (46.7%), shortness of breath (44.9%), and fatigue (44.9%) were the most common symptoms at presentation (Supplementary Figure 2). The mean interval from symptom onset to hospital presentation was 5 ± 3.1 days, and 20.4% of patients had ≤ 93% baseline oxygen saturation. All patients included in this study had non-severe COVID-19 (WHO scores of 3–5) on admission. On chest computed tomography (CT) examinations, 59.9% of patients showed ≤ 25% lung involvement, 35.3% showed 26–50% involvement, and 4.8% showed >50% lung involvement. Thirty-six (21.6%) cases presented criteria for significant pulmonary involvement (oxygen saturation ≤ 93% and/or >50% lung involvement on CT) at admission (Supplementary Table 2).

### Primary Outcome

Worst WHO clinical improvement scores during hospitalization were 3 (mild disease) in 81 (48.5%; 95% CI, 41.0–56.1%) cases, 4 or 5 (moderate disease) in 73 (43.7%; 95% CI, 36.3–51.3%) cases, and 6–8 (severe disease) in 13 (7.8%; 95% CI, 4.4–12.6) cases (Supplementary Table 3). Progression to severe disease was associated with obesity (*p* = 0.003), diabetes (*p* < 0.001), and oxygen saturation (*p* = 0.001) and lung involvement (*p* = 0.001) on admission, but not with age or sex (Table 1).

**Table 1:** Baseline patient characteristics by primary outcome ^*^.

**Clinical Conditions**	**Total**	**Score 3–5 (*n* = 154/167**)	**Score 6–8 (*n*= 13/167)**	**Fisher's exact test *P*-value**
	***n***	***n*** **(%)**	***n*** **(%)**	
**Sex**				
Male	110	99 (90.0)	11 (10.0)	0.22
Female	57	55 (96.5)	2 (3.5)	
**Age**				
<60 years old	114	105 (92.1)	9 (7.9)	1.00
60 and older	53	49 (92.5)	4 (7.5)	
**Signs of pulmonary involvement**				
O_2_ sat > 93% and CT ≤ 50% ^†^	131	126 (96.2)	5 (3.8)	0.001
O_2_ sat ≤ 93% or CT > 50%	36	28 (77.8)	8 (22.2)	
**Obesity**				
No (BMI < 30 kg/m^2^) ^†^	79	78 (98.7)	1 (1.3)	0.003
Yes (BMI ≥ 30 kg/m^2^)	88	76 (86.4)	12 (13.6)	
**Diabetes**				
No	126	123 (97.6)	3 (2.4)	< 0.001
Yes	41	31 (75.6)	10 (24.4)	
**Asthma/COPD**				
No	164	151 (92.1)	13 (7.9)	1.00
Yes	3	3 (100.0)	0 (0.0)	
**Dyslipidemia**				
No	138	128 (92.8)	10 (7.2)	0.70
Yes	29	26 (89.7)	3 (10.3)	
**Coronary disease**				
No	163	151 (92.6)	12 (7.4)	0.28
Yes	4	3 (75.0)	1 (25.0)	

* *Highest modified World Health Organization WHO Ordinal Scale for Clinical Improvement*.

† *Extent of lung involvement on CT examination*.

*BMI, body mass index; O_2_ sat, oxygen saturation; COPD, chronic obstructive pulmonary disease; CT, computed tomography*.

### Secondary Outcomes

The mean hospital LOS was 9.1 ± 6.9 days. In total, 119 patients were admitted to the ICU; the mean ICU LOS was 7.6 ± 6.8 days (Supplementary Table 4). According to the report on the BRACE-CORONA trial (13), the mean numbers of days alive and out of hospital did not differ among patients hospitalized with mild to moderate COVID-19 according to ACEI/ARB discontinuation or continuation.

**Figure 1:**
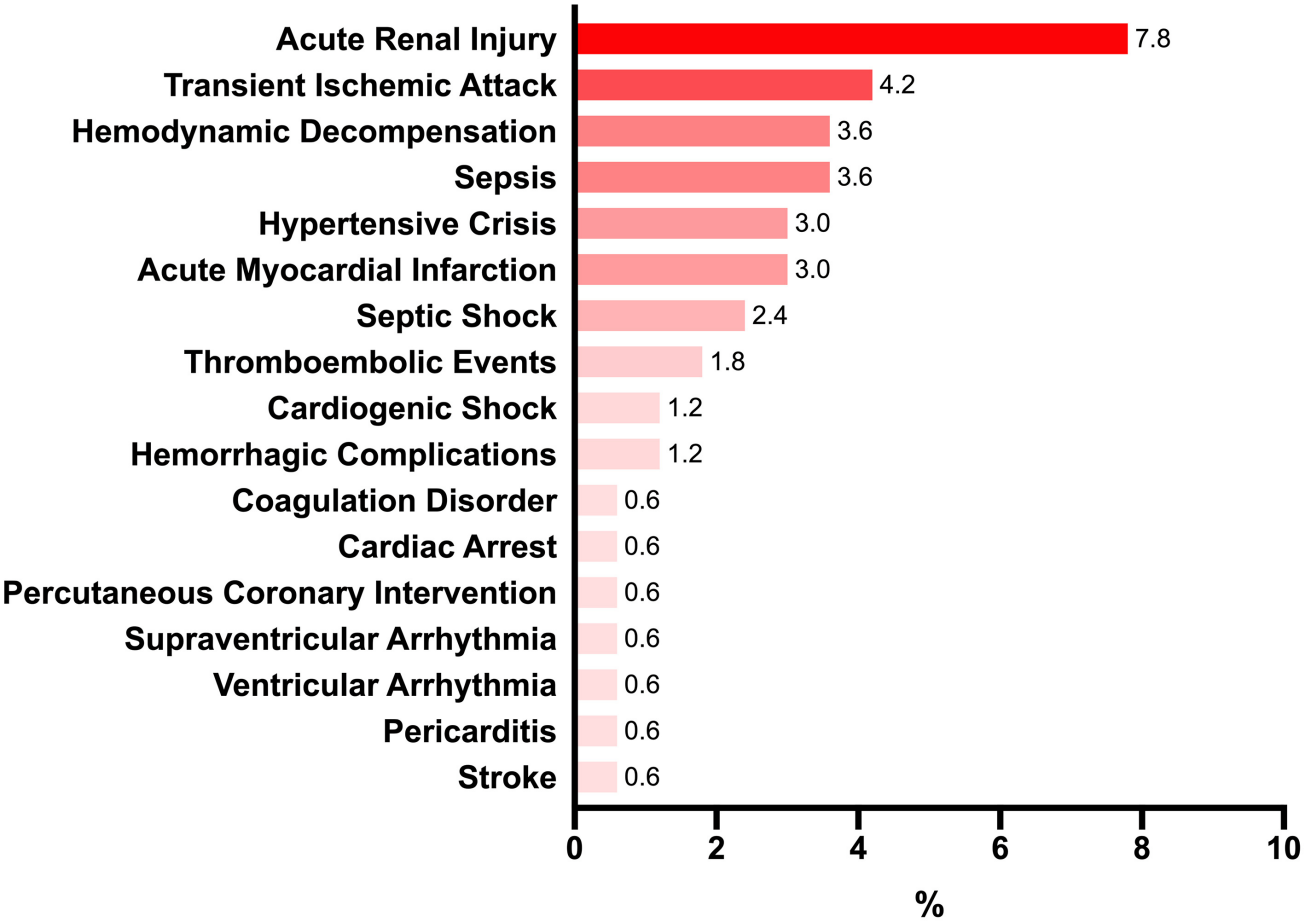
Main complications occurring during hospitalization (%).

At least one complication occurred during hospitalization in 29 (17.4%) patients. The number of complications per patient ranged from one to nine. The most common complication was acute renal injury [*n* = 13 (7.8%); Figure 1]. The criteria used for the identification of these complications have been provided in the BRACE-CORONA trial (13). The most commonly administered treatments were antibiotics (98.2%), anticoagulants (68.3%), and corticosteroids (59.9%; Supplementary Figure 3).

### Biomarkers

Blood samples were collected a mean of 2.8 days after hospitalization. Levels of IL-10, IP-10, G-CSF, IFN- α2, IL-1ra, IL-15, IL-1α, IL-12 (p70), IL-2, IL-17A, GM-CSF, IL-8, IL-6, MCP-1, and CRP were higher in patients with severe than in those with non-severe disease. In contrast, levels of lymphocytes and MIP-1β were lower in patients with severe than in those with non-severe disease (Table 2). IL-3 and IL-4 were not detected in >50% of patients and were excluded from further analyses.

**Table 2:** Biomarker levels according to WHO Ordinal Scale for Clinical Improvement.

	**All**			**Non-severe (score 3–5)**			**Severe (score 6–8)**		
	**Median**	**Mean**	**SD**	**Median**	**Mean**	**SD**	**Median**	**Mean**	**SD**
**Lymphocytes** ^*^	1.40	1.51	0.74	1.44	1.53	0.65	0.73	1.30	1.45
**Monocytes** ^*^	0.23	0.36	0.38	0.23	0.35	0.32	0.10	0.53	0.82
**CRP** ^†^	2.23	4.53	5.28	2.08	4.05	4.95	10.70	10.20	6.01
**MIP-1β**	21.8	22.5	11.1	22.0	22.9	11.0	13.8	17.6	10.5
**VEGF**	57.2	80.5	83.9	55.0	81.1	86.8	67.4	72.8	34.6
**TNF-β**	0.9	27.1	102.1	0.8	28.9	106.1	2.6	6.4	11.1
**TNF-α**	15.8	16.0	7.4	15.6	15.8	7.4	18.5	18.1	6.6
**MIP-1α**	5.1	7.3	11.0	5.1	7.3	11.4	7.6	7.4	5.1
**MCP-1**	466	584	428	435	573	435	792	721	316
**IP-10**	2,357	2,765	2,482	1,842	2,509	2,155	5,434	5,798	3,904
**IL-17A**	0.4	2.7	5.9	0.2	2.6	6.0	2.0	4.3	5.2
**IL-15**	5.1	6.1	4.8	5.1	5.9	4.8	9.2	9.2	3.9
**IL-13**	1.7	13.1	43.0	1.3	13.8	44.6	1.7	4.7	11.4
**IL-12 (p70)**	0.8	1.4	2.1	0.6	1.4	2.0	1.6	2.5	2.3
**IL-12 (p40)**	1.3	4.2	6.6	1.2	4.1	6.6	4.3	6.3	6.2
**IL-10**	15.5	26.3	29.4	13.4	23.2	25.2	43.8	62.0	49.2
**IL-8**	8.9	14.2	18.5	8.6	13.8	18.5	13.9	18.8	19.2
**IL-7**	7.7	10.0	12.7	7.0	10.1	13.2	9.1	9.1	5.0
**IL-6**	5.5	28.4	76.8	5.1	28.1	78.8	12.6	32.1	49.3
**IL-5**	1.5	6.4	19.1	1.4	6.6	19.8	2.0	4.3	6.8
**IL-4**	0.0	239	916	0.0	254	951	10	62	185
**IL-3**	0.0	0.06	0.11	0.0	0.05	0.11	0.13	0.12	0.08
**IL-2**	0.48	1.11	2.01	0.29	1.06	2.03	1.15	1.72	1.71
**IL-1ra**	52	112	196	48	91	138	231	358	464
**IL-1β**	0.58	1.25	2.08	0.58	1.26	2.15	0.83	1.12	0.93
**IL-1α**	25.4	59.6	137.8	23.6	59.9	143.1	48.7	55.9	39.0
**IFN-γ**	5.5	12.6	22.5	5.4	12.5	23.2	11.9	13.4	11.2
**IFN-α2**	28.2	40.8	54.2	26.4	37.8	54.1	74.1	75.7	43.2
**GM-CSF**	2.68	4.01	5.00	1.92	3.84	5.09	5.37	5.97	3.37
**G-CSF**	57.8	65.7	55.4	53.4	61.3	52.7	118.6	118.3	61.2
**eotaxin**	177	195	96	177	196	99	182	186	51
**EGF**	208	247	205	204	251	211	214	197	111

*CRP, C-reactive protein; EGF, epidermal growth factor; G-CSF, granulocyte colony-stimulating factor; GM-CSF, granulocyte-macrophage colony-stimulating factor; IFN, interferon; IL, interleukin; IP-10, interferon gamma-induced protein 10; MCP-1, monocyte chemoattractant protein 1; MIP, macrophage inflammatory protein; SD, standard deviation; TNF, tumor necrosis factor; VEGF, vascular endothelial growth factor; WHO, World Health Organization*.

* *10^9^ cells/L*,

† *mg/L; all other biomarker units are pg/mL*.

Fifteen cytokines were found to be useful for the prediction of progression to severe COVID-19 [areas under the ROC curve (AUCs), 0.667–0.836]. Increased levels of 14 cytokines and decreased levels of MIP-1β were associated with COVID-19 severity. AUCs for IL-10, IP-10, G-CSF, IFN- α2, IL-1ra, and IL-15 were ≥0.75 (Table 3). Increased CRP levels and reduced lymphocyte counts were also associated with disease severity (AUCs, 0.825 and 0.742, respectively; Supplementary Figure 4).

**Table 3:** Distinction of severe (modified WHO score 6–8) and non-severe (modified WHO score 3–5) cases by areas under ROC curves.

**Biomarkers**	**Area under curve**	***P*** **-value**	**Cut-off for 90% sensitivity**
**IL-10**	**0.836**	**<0.001**	**26.0**
**CRP** ^†^	**0.825**	** <0.001**	**2.70**
**IP-10**	**0.812**	**<0.001**	**2400**
**G-CSF**	**0.788**	**0.001**	**54.0**
**IFN-α2**	**0.775**	**0.001**	**19.4**
**IL-1ra**	**0.759**	**0.002**	**29.5**
**IL-15**	**0.750**	**0.003**	**5.1**
**Lymphocytes** ^*^ ^§^	**0.742**	**0.004**	**2.11**
**IL-1α**	**0.731**	**0.006**	**26.1**
**IL-12 (p70)**	**0.730**	**0.006**	**0.91**
**IL-2**	**0.715**	**0.010**	**0.35**
**IL-17A**	**0.711**	**0.012**	**0.21**
**GMCSF**	**0.710**	**0.012**	**2.69**
**MIP-1**β ^§^	**0.686**	**0.026**	**32.6**
**IL-8**	**0.682**	**0.030**	**6.2**
**IL-6**	**0.678**	**0.033**	**2.6**
**MCP-1**	**0.667**	**0.045**	**406**
IL-12 (p40)	0.664	0.051	^#^
TNF-β	0.644	0.085	^#^
TNF-α	0.607	0.202	^#^
IFN-γ	0.607	0.200	^#^
IL-1β	0.601	0.225	^#^
MIP-1α	0.584	0.313	^#^
IL-5	0.581	0.330	^#^
IL-7	0.576	0.366	^#^
VEGF	0.564	0.441	^#^
IL-13	0.539	0.637	^#^
Eotaxin	0.503	0.971	^#^
EGF	0.473	0.743	^#^
Monocytes ^*^	0.378	0.143	^#^

*CRP, C-reactive protein; EGF, epidermal growth factor; G-CSF, granulocyte colony-stimulating factor; GM-CSF, granulocyte-macrophage colony-stimulating factor; IFN, interferon; IL, interleukin; IP-10, interferon gamma-induced protein 10; MCP-1, monocyte chemoattractant protein 1; MIP, macrophage inflammatory protein; ROC, receiver operating characteristic; SD, standard deviation; TNF, tumor necrosis factor; VEGF, vascular endothelial growth factor; WHO, World Health Organization*.

* *10^9^ cells/L*,

† *mg/L; all other biomarker units are pg/mL*;

§ *In contrast to other biomarkers, reduced values are predictors of disease progression; Bold values indicate significance at p < 0.05*.

# *Not calculated due to lack of statistical association at established value*.

### Predictive Model

The initial model for the prediction of the risk of progression of COVID-19 included diabetes, obesity, hypoxemia, lung involvement on CT, the CRP level, the lymphocyte count, and levels of 15 cytokines. Five variables were selected automatically in a forward stepwise manner: the IL-10 level (>26.0 pg/mL), diabetes, the IL-12 (p70) level (>0.91 pg/mL), obesity, and significant lung involvement on admission (oxygen saturation ≤ 93% or >50% lung involvement on CT). The IL-10 level was associated strongly with disease severity [odds ratio (OR) = 32]. The ORs for the other four variables also showed associations with progression to severe disease (Table 4), and the predictive value of the model increased strongly with the addition of these variables (OR = 78.3). The ROC curve for the predictive model showed a very high discriminatory power between the two groups with an AUC of 0.981 (Supplementary Figure 5).

**Table 4:** Forward stepwise logistic regression results for COVID-19 severity.

**Variables in the equation**		**β**	***P*** **-value**	**Odds ratio**
Step 1	IL-10 > 26	3.47	0.001	32.0
Step 2	Diabetes	2.82	< 0,001	16.8
	IL-10 > 26	3.68	0.001	39.6
Step 3	IL-12 (p70) > 0.91	3.30	0.009	27.1
	Diabetes	3.54	0.000	34.3
	IL-10 > 26	3.89	0.002	49.1
Step 4	Obesity	2.86	0.046	17.5
	IL-12 (p70) > 0.91	3.33	0.024	28.0
	Diabetes	3.89	0.001	49.0
	IL-10 > 26	4.35	0.002	77.2
Step 5 (final model) ^*^	Lung involvement ^†^	2.16	0.045	8.7
	Obesity	3.81	0.032	45.2
	IL-12p70 > 0.91	3.89	0.026	49.0
	Diabetes	3.58	0.004	35.9
	IL-10 > 26	4.36	0.005	78.3

*IL, interleukin*.

* *Constant = −14.15 and Hosmer–Lemeshow test p = 1.000*.

† *Significant lung involvement on admission (oxygen saturation ≤93% or >50% lung involvement on computed tomography examination)*.

*Biomarker values are presented in pg/mL*.

**Figure 2:**
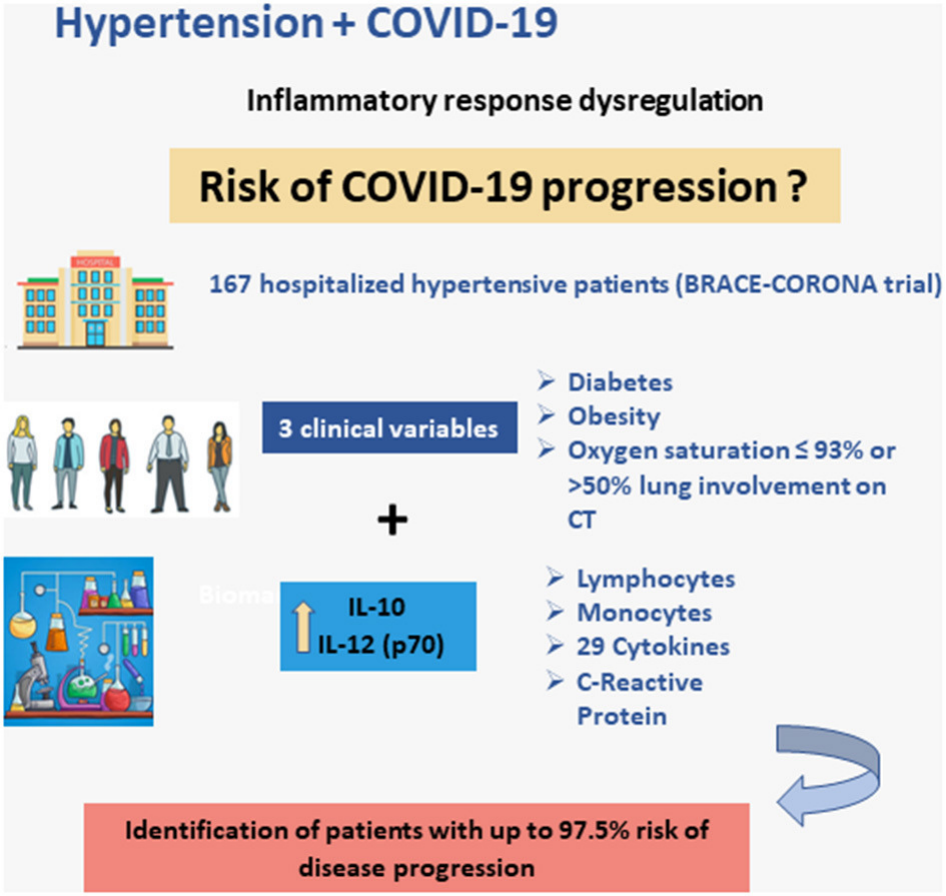
Schematic illustration of the main findings: model for the prediction of the risk of progression of COVID-19 including three clinical variables and two biomarkers: IL-10 and IL-12 (p70).

In the presence of two or three clinical comorbidities, the predictive capability of these biomarkers increased markedly (Figure 2). In patients with diabetes and obesity, for example, the likelihood of disease progression increased from 0.1% with low IL-10 and IL-12 (p70) levels to >80% with levels of these cytokines exceeding the 90% sensitivity thresholds. Similarly, the risk of progression to severe disease in the presence of three clinical comorbidities was 1.0% with IL-10 and IL-12 (p70) levels below the thresholds and 97.5% with levels exceeding the thresholds (Table 5).

**Table 5:** Probability of COVID-19 progression according to the final logistic model.

	**IL-10 ≤ 26 and IL12p70 ≤ 0.91**	**IL10 ≤ 26 and IL12p70 > 0.91**	**IL10 > 26 and IL12p70 ≤ 0.91**	**IL10 > 26 and IL12p70 > 0.91**
No clinical risk factors ^†^	<0.1%	<0.1%	<0.1%	0.3%
Only diabetes	<0.1%	0.1%	0.2%	8.9%
Only obesity	<0.1%	0.2%	0.3%	10.9%
Only significant lung involvement ^‡^	<0.1%	<0.1%	<0.1%	2.3%
Diabetes + obesity	0.1%	5.3%	8.3%	81.5%
Diabetes + significant lung involvement	< 0.1%	1.1%	1.7%	45.9%
Obesity + significant lung involvement	< 0.1%	1.3%	2.1%	51.6%
Diabetes + obesity + significant lung involvement	1.0%	32.8%	43.9%	97.5%

*IL, interleukin. Biomarker values are presented in pg/mL*.

† *Clinical risk factors in the model are diabetes, obesity, and significant lung involvement on admission*.

‡ *Oxygen saturation ≤ 93% or >50% lung involvement on computed tomography examination*.

## Discussion

In this study, we analyzed immune response patterns, including levels of 29 cytokines, CRP, monocytes, and lymphocytes, in a large sample (*n* = 167) of hospitalized hypertensive patients from the BRACE-CORONA trial (13). In univariate analysis, progression to severe COVID-19 was associated with clinical factors (diabetes, obesity, and lung involvement on admission) and levels of biomarkers, including 15 cytokines, CRP, and lymphocytes. We propose a logistic regression model that includes clinical variables (diabetes, obesity, and significant lung involvement) and critical biomarkers [IL-10 and IL-12 (p70)]. This combined use of clinical risk factors and biomarkers for the prediction of COVID-19 severity at admission is novel.

Clinical comorbidities, particularly diabetes, hypertension, and other cardiovascular diseases, have been associated with COVID-19 severity, as they are more prevalent in non-survivors and patients requiring ICU care (1, 30–32). However, the mechanisms involved in the increased risk of COVID-19 in these patients are not understood completely. Infections are more prevalent and have more complicated courses in patients with diabetes, possibly due to disturbances in humoral and cellular immunity and exaggerated pro-inflammatory cytokine responses (33, 34). In addition, obesity has been associated with ICU admission and mortality in patients with COVID-19, which may be related to the presence of angiotensin-converting enzyme 2 receptors in adipose tissue, elevated pro-inflammatory cytokine levels, increased susceptibility to infection by various pathogens (35), and pro-coagulant profiles (36). Moreover, the extent of CT lung involvement has been correlated with COVID-19 severity, and severity scores for chest CT findings have been proposed to enable the differentiation of clinical forms and prediction of clinical outcomes (37, 38). The lack of association between age and the outcome in the present study may be related to the relative young mean age of our sample, due to the exclusion of patients with severe disease in the first 24 h after admission.

In this study, admission levels of 17 biomarkers (increased levels of CRP and 14 cytokines and reduced levels of MIP-1β and lymphocytes) were associated significantly with progression to severe disease. Our biomarker findings are similar to previously reported associations of the levels of several cytokines (e.g., IL-1ra, IL-2, IL-6, IL-8, IL-10, and IP-10) with COVID-19 severity and mortality (25, 27, 39–41). The association of the IL-10 level with COVID-19 progression to severity has been reported in a considerable number of publications (40–42). IL-10 is an immunoregulatory cytokine with the main functions of limiting inflammatory responses and regulating immune cell differentiation and proliferation (43). Information about the role of IL-12 (p70) in COVID-19 is more limited. Consistent with our findings, higher levels of IL-12 (p70) have been associated with severe COVID-19 (44, 45). IL-12 is a heterodimeric cytokine composed of p35 and p40 subunits that enhances connections between the innate and adaptive immune responses; its expression is induced via a pathogen-associated molecular response when a virus enters a cell (46).

Although the ability of clinical and laboratory variables to independently predict COVID-19 severity has been assessed extensively and predictive models have been proposed, no definitive prognostic biomarker or effective predictive model for the identification, at the time of hospital admission, of patients who will require ICU care, mechanical ventilation, or inotropic support has emerged (47, 48). According to the model we propose, the probability of progression to severe disease in hypertensive patients with obesity and diabetes is 0.1% in the absence of increased IL-10 and IL-12 (p70) levels, but 81.5% with levels of these two cytokines exceeding the 90% sensitivity thresholds. Similarly, in the presence of the three clinical comorbidities (obesity, diabetes, and oxygen saturation ≤ 93% or >50% lung involvement on CT), the probability of progression is 1% with lower IL-10 and IL-12 (p70) levels, but 97.5% with elevated levels of these biomarkers. A practical approach to model application for the estimation of the risk of progression to severe COVID-19 would be to measure IL-10 and IL-12 (p70) levels on admission in hypertensive patients with two or three of the relevant clinical comorbidities.

### Limitations

This study has some limitations. Blood samples were collected a mean of 2.8 days after hospitalization (usually within 24 h after confirmation of SARS-CoV-2 infection); with a median 6-day interval between symptom onset and hospital admission, and our population included only hypertensive patients who were taking ACEi or ARBs, which might limit the generalizability of our results. Nevertheless, we believe that the widespread use of these drugs in the hypertensive population associated with the multicentric nature of the study might help to ensure a good external validity. Besides, we were not able to validate our model with a different patient sample. Additional studies are needed to validate the results obtained here in more heterogeneous populations of hypertensive patients and also to evaluate the applicability of the proposed model in non-hypertensive COVID-19 populations.

## Conclusion

The measurement of IL-10 and IL-12 (p70) levels on admission may be useful for the identification of hypertensive patients at greater risk of COVID-19 progression, particularly in the presence of classical clinical comorbidities (obesity, diabetes, and extensive lung involvement). We propose a new biomarker-based approach to improve the prediction of COVID-19 progression in hypertensive patients, which may help physicians identify patients at high risk who would benefit from more intensive surveillance and treatment.

## Data Availability Statement

The raw data supporting the conclusions of this article will be made available by the authors, without undue reservation.

## Ethics Statement

The studies involving human participants were reviewed and approved by Brazilian Ministry of Health's National Commission for Research Ethics (CAAE # 30432020.2.0000.5249). The patients/participants provided their written informed consent to participate in this study.

## Author Contributions

RM-B, EM, AS, FB, and RRL: study design. OS, AM, RDL, AF, GA, DA, and MS: patient recruitment, data, and sample collection organization. RM-B and EM: application for the funding and writing-original draft preparation. AM, AF, GA, TP, TF, VL, KG, NO, FD, MK, and RD: patient recruitment and sample collection. NV, LM, MC, and PP-C: biomarker processing and analyses. RDL, RM-B, AM, and RRL: data curation. RRL: statistical analyses. AS, FB, and RDL writing-review and editing. All authors contributed to the article and approved the submitted version.

## Conflict of Interest

RDL reports receiving grant support from Bristol-Myers Squibb, GlaxoSmithKline, Medtronic, Sanofi, and Pfizer, and consulting fees from Bayer, Boehringer Ingelheim, Bristol-Myers Squibb, Daiichi-Sankyo, GlaxoSmithKline, Medtronic, Merck, Pfizer, Sanofi, and Portola. AM reports receiving consulting fees from Pfizer, Bayer, AstraZeneca, Novartis, Daiichi-Sankyo, Zodiac, Roche, and Janssen. AF reports receiving consulting fees from Pfizer, Bayer, Daiichi-Sankyo, Boehringer, and Servier. GA reports receiving consulting fees from Bayer, Pfizer, Servier, AstraZeneca, and Daichii Sankyo. DA reports receiving consulting fees from Boehringer Ingelheim, AstraZeneca, Bayer, and Servier. OS reports receiving grant support from Boehringer Ingelheim and consulting fees from Pfizer, Bayer, Daiichi-Sankyo, and Boehringer Ingelheim. The remaining authors declare that the research was conducted in the absence of any commercial or financial relationships that could be construed as a potential conflict of interest.

## Publisher's Note

All claims expressed in this article are solely those of the authors and do not necessarily represent those of their affiliated organizations, or those of the publisher, the editors and the reviewers. Any product that may be evaluated in this article, or claim that may be made by its manufacturer, is not guaranteed or endorsed by the publisher.

## Abbreviations

ACEI: angiotensin-converting enzyme inhibitor
ARB: angiotensin receptor blocker
AUC: area under the receiver operating characteristic curve
CI: confidence interval
CRP: C-reactive protein
CT: computed tomography
G-CSF: granulocyte colony-stimulating factor
GM-CSF: granulocyte-macrophage colony-stimulating factor
ICU: intensive care unit
IFN: interferon
IL: interleukin
IP-10: interferon gamma–induced protein 10
LOS: length of stay
MCP-1: monocyte chemoattractant protein-1
MIP: macrophage inflammatory protein
OR: odds ratio
TNF: tumor necrosis factor
WHO: World Health Organization.
