# Supplementary material for: IL-10 and IL-12 (P70) Levels Predict the Risk of Covid-19 Progression in Hypertensive Patients: Insights From the BRACE-CORONA Trial
Source: Front Cardiovasc Med. 2021 Jul 27;8:702507. doi: 10.3389/fcvm.2021.702507 (PMC8353079; doi:10.3389/fcvm.2021.702507)
Supplement: Supplementary file 1 [file Data_Sheet_1.PDF]

Supplementary Material

1. Supplementary Data

<u>Inclusion criteria:</u>

- Patients aged ≥18 years hospitalized with a confirmed diagnosis of COVID-19 under use of angiotensin receptor blockers or angiotensin converting enzyme inhibitors.
- The patient (or legal representative) must be able to give informed consent in accordance with ICH GCP guidelines and local legislation and/or regulations.

<u>Exclusion criteria</u>

- Hospitalization due to decompensated heart failure in the last 12 months
- Use of more than 3 anti-hypertensive drugs
- Use of Sacubitril/Valsartan
- Patients under mechanical ventilation
- Hemodynamic instability in the first 24 hours until the moment of confirmed diagnosis of COVID-19; acute renal failure; shock

2. Supplementary Tables and Figures

2.1 Supplementary Tables

Supplementary Table 1. Primary outcome according to modified WHO Ordinal Scale for Clinical Improvement

Patient State	Descriptor	Score
**Uninfected**	No clinical or virological evidence of infection	**0**
**Ambulatory**	No limitation of activities	**1**
	Limitation of activities	**2**
**Hospitalized Mild disease**	Hospitalized, no oxygen therapy	**3**
	Oxygen by mask or nasal prongs	**4**
**Hospitalized Severe disease**	Non-invasive ventilation or high-flow oxygen	**5**
	Intubation and mechanical ventilation	**6**
	Ventilation + vasopressors, dialysis, or ECMO	**7**
**Dead**	Death	**8**

ECMO indicates extracorporeal membrane oxygenation; WHO, World Health Organization. Modified from World Health Organization. WHO R&D blueprint: COVID-19 Therapeutic Trial Synopsis. [29]

Supplementary Table 2. Signs of pulmonary involvement upon admission according to oxygen saturation and lung involvement on computed tomography

CT scan*	Total	Oxygen saturation	
		≤ 93 %	>93%
	n (%)	n (%)	n (%)
**≤ 25%**	100 (59.9)	13 (7.8)	87 (52.1)
**26 to 50%**	59 (35.3)	15 (9.0)	44 (26.3)
**> 50%**	8 (4.8)	6 (3.6)	2 (1.2)
**Total**	167 (100.0)	34 (20.4)	89 (53.3)

CT scan indicates computed tomography examination.

* Percentage of lung involvement on computed tomography examination

Supplementary Table 3. Severity of disease according to WHO score.

WHO score	Frequencies		
	Original scale	Grouped scale	
	n (%)	n (%)	95% CI
**3** (hospitalized with no oxygen)	81 (48.5)	**81 (48.5)**	41.0 - 56.1%
**4** (hospital + oxygen, no ventilatory support)	61 (36.5)	**73 (43.7)**	36.3 - 51.3%
**5** (hospital + non-invasive ventilatory support)	12 (7.2)		
**6** (hospital+ MV)	0 (0.0)	**13 (7.8)**	4.4 - 12.6%
**7** (hospital + MV + inotropic support)	10 (6.0)		
**8** (death)	3 (1.8)		
Total	167 (100.0)	167 (100.0)	

CI indicates confidence interval; hospital, hospitalization; MV: mechanical ventilatory support; WHO, World Health Organization

Supplementary Table 4. Length of stay (LOS) in hospital and ICU

	WHO score	Descriptive statistics			
		**Mean (SD)**	**Minimum**	**Median**	**Maximum**
Days of symptoms*	Total (n = 167)	5.7 (3.1)	0.0	5.0	17.0
	Score 3 (n = 81)	5.3 (3.0)	1.0	5.0	17.0
	Score 4 ou 5 (n = 73)	6.4 (3.2)	0.0	7.0	14.0
	Scores 6 a 8 (n = 13)	4.6 (2.4)	0.0	4.0	9.0
LOS in hospital	Total (n =167)	9.1 (6.9)	2.0	7.0	33.0
	Score 3 (n = 81)	6.1 (2.9)	2.0	6.0	19.0
	Score 4 ou 5 (n = 73)	9.4 (4.8)	3.0	8.0	31.0
	Scores 6 a 8 (n = 13)	27.2 (6.7)	12.0	31.0	33.0
LOS in ICU	Total (n = 119) †	7.6 (6.8)	2.0	5.0	34.0
	Score 3 (n = 50)	4.1 (1.9)	2.0	4.0	12.0
	Score 4 ou 5 (n = 56)	7.3 (4.8)	2.0	6.0	28.0
	Scores 6 a 8 (n = 13)	22.5 (6.8)	11.0	23.0	34.0

ICU indicates intensive care unit; LOS, length of stay; SD: standard deviation.

* pre-hospitalization; † 48 patients (28.7%) did not stay in ICU

2.1 Supplementary Figures

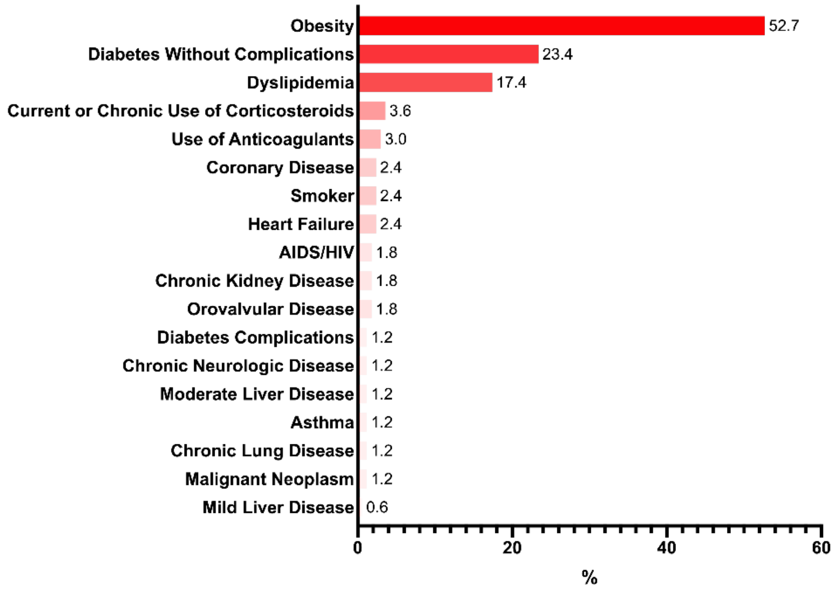

Supplementary Figure 1: Comorbidities at admission (%).

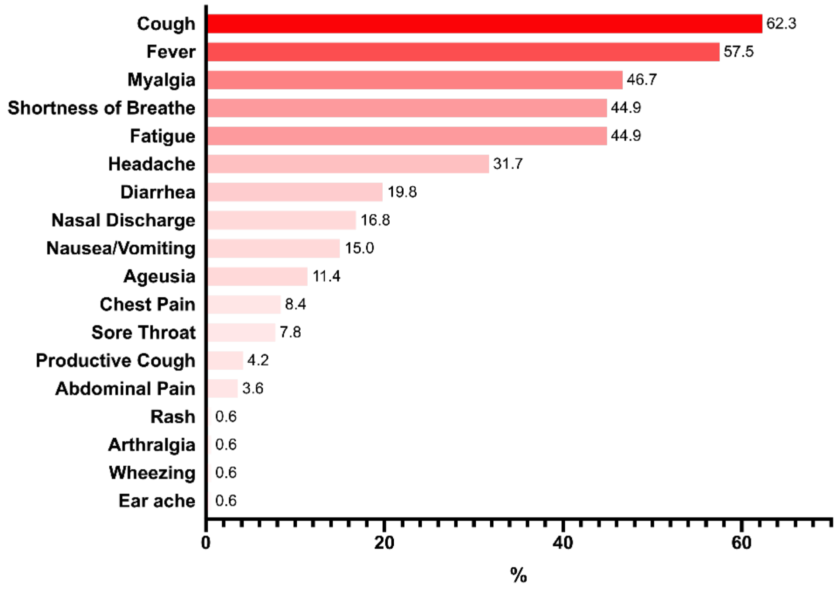

Supplementary Figure 2: Symptoms at presentation (%).

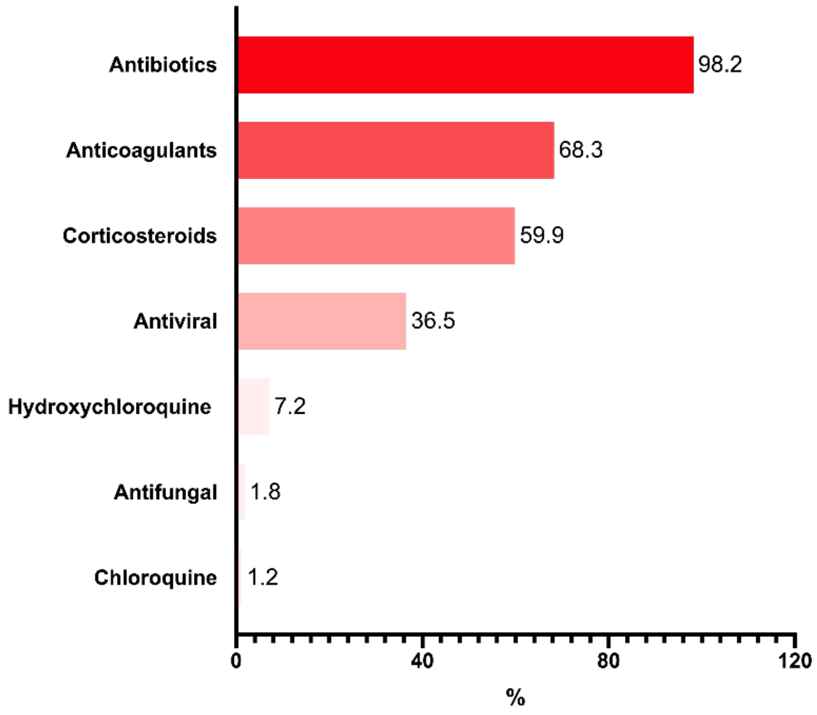

Supplementary Figure 3: Treatment during hospitalization (%).

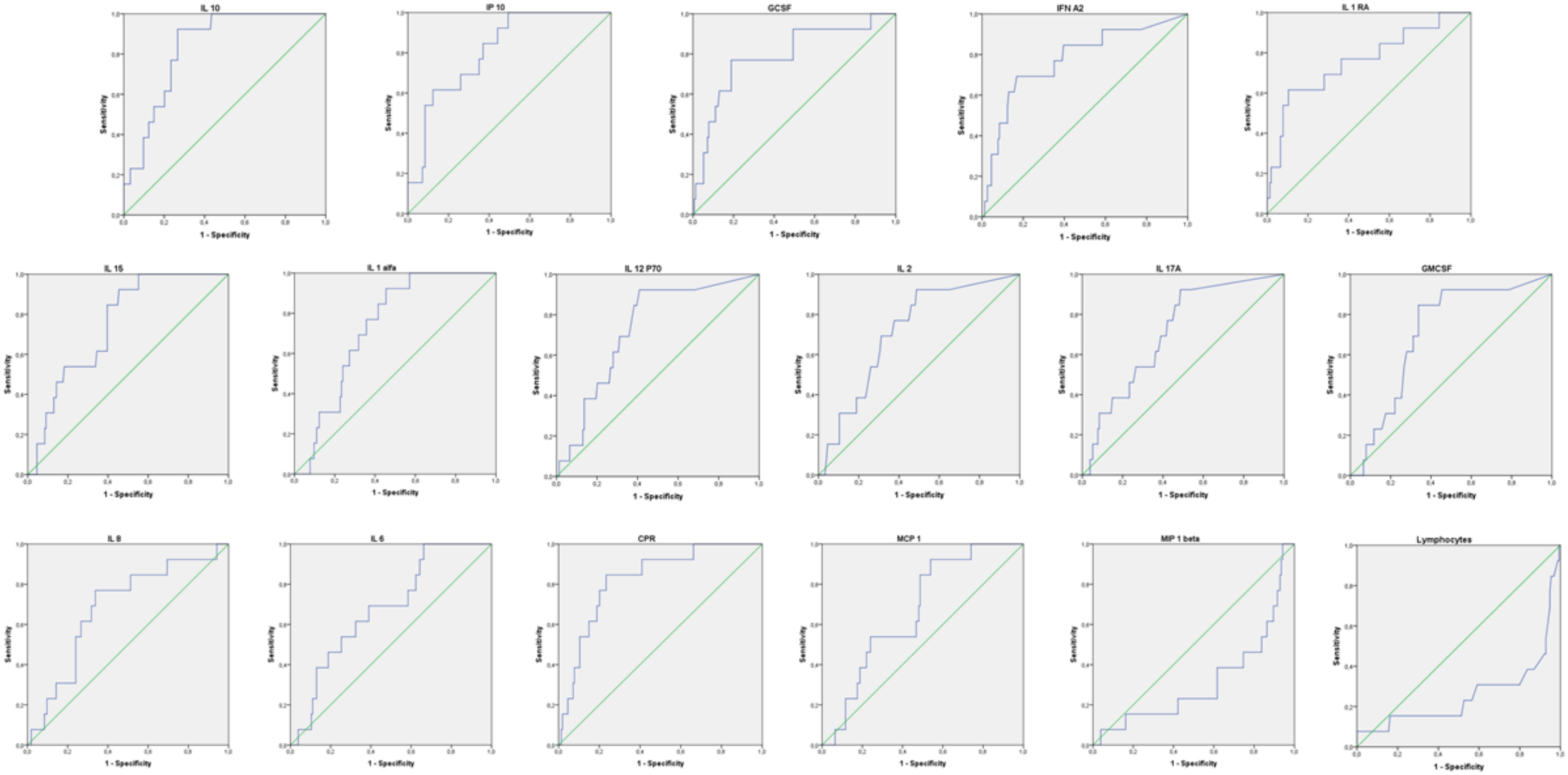

Supplementary Figure 4: Receiver Operating Characteristic curves for 15 cytokines, C-reactive protein and lymphocytes.

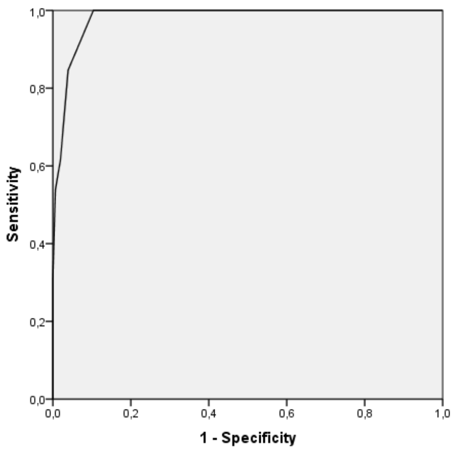

Supplementary Figure 5: Receiver Operating Characteristic curves for the predictive model
